# Effects of music-based interventions on rehabilitation outcomes after stroke: a systematic review and meta-analysis of randomized controlled trials

**DOI:** 10.3389/fneur.2026.1907080

**Published:** 2026-08-28

**Authors:** Delin Liu, Tengteng Kang, Yakun Liang, Cunzhe Tong, Yufen Xue

**Affiliations:** 1 Department of Music and Dance, Zhoukou Normal University, Zhoukou, Henan, China; 2 Department of Nursing, Zhoukou Vocational and Technical College, Zhoukou, China; 3 Advanced Medical and Dental Institute, Universiti Sains Malaysia, Penang, Malaysia; 4 School of Nursing and Health, Henan University, Kaifeng, Henan, China

**Keywords:** meta-analysis, music-based interventions, rehabilitation, stroke, systematic review

## Abstract

**Objective:**

To systematically evaluate the effects of music-based interventions on rehabilitation outcomes in patients with stroke and provide evidence for clinical practice.

**Methods:**

Web of Science, PubMed, Embase, Cochrane Library, Scopus, ProQuest, CINAHL, SinoMed (CBM), China National Knowledge Infrastructure (CNKI), Wanfang Database, and Technology Journal Database (VIP) were searched from inception to April 7, 2026. Randomized controlled trials comparing music-based interventions plus conventional rehabilitation with conventional rehabilitation alone in patients with stroke were included. Literature screening, data extraction, and quality assessment were independently conducted by two reviewers. Meta-analysis was performed using Review Manager (RevMan) 5.4.

**Results:**

A total of 21 randomized controlled trials involving 1,029 patients with stroke were included. Compared with conventional rehabilitation, music-based interventions significantly improved upper limb motor function [SMD = 0.58, 95% CI (0.26, 0.91), *p* = 0.0004], gait speed [SMD = 0.89, 95% CI (0.16, 1.62), *p* = 0.02], balance [SMD = 0.45, 95% CI (0.17, 0.74), *p* = 0.002], lower limb motor function [SMD = 0.99, 95% CI (0.59, 1.39), *p* < 0.001], activities of daily living [SMD = 0.88, 95% CI (0.68, 1.08), *p* < 0.001], and quality of life [SMD = 0.31, 95% CI (0.13, 0.49), *p* = 0.0009].

**Conclusion:**

Music-based interventions appear to be an effective adjunct to conventional stroke rehabilitation. They may improve motor function, gait and balance performance, activities of daily living, and quality of life in patients with stroke. Further large-scale, high-quality randomized controlled trials are warranted to confirm these findings.

**Systematic review registration:**

https://www.crd.york.ac.uk/PROSPERO/view/CRD420261419159.

## 1 Introduction

Stroke is one of the leading causes of death and long-term disability among adults worldwide (1). Approximately 70% of survivors are left with varying degrees of motor impairment, cognitive impairment, and psychological issues, with hemiplegia being the most common sequela (2). Gait abnormalities and upper limb dysfunction are particularly prominent, manifesting as slowed walking speed, reduced stride length, increased asymmetry, and decreased upper limb coordination, which impair patients’ ability to perform activities of daily living and their quality of life (3). In addition, post-stroke cognitive impairment, mood disorders, swallowing disorders, and aphasia are also common and significantly impact rehabilitation progress and quality of life (4–6). Although conventional rehabilitation therapies such as neurodevelopmental therapy, physical therapy, and occupational therapy have been widely used in stroke rehabilitation, their effectiveness may be limited. Patients often experience fatigue and apathy due to monotonous training and slow progress, leading to reduced treatment adherence (7). Therefore, exploring adjunctive therapeutic approaches that can enhance rehabilitation outcomes and increase patient engagement holds significant clinical importance.

As non-pharmacological and non-invasive rehabilitation approaches, music-based interventions have received increasing attention in stroke rehabilitation in recent years (8). Music-based interventions include passive approaches, such as music listening, and active approaches, such as rhythmic auditory stimulation, music-supported therapy, and instrument-based training (9). Among these, rhythmic auditory stimulation uses rhythmic auditory cues to guide the temporal activity of the motor system, improving gait parameters through auditory-motor coupling mechanisms (10). On the other hand, music-supported therapy enhances upper limb motor skills and coordination through the playing of keyboard and percussion instruments (11). Music-to-sound conversion technology transforms the patient’s movements into auditory feedback, aiding in sensorimotor integration and pain relief (12). Additionally, personalized music listening has been applied to improve post-stroke cognitive impairments by promoting cognitive recovery through the evocation of autobiographical memories and the enhancement of default mode network connectivity (13). Previous randomized controlled trials have reported that rhythmic auditory stimulation may improve walking speed, stride length, cadence, and gait symmetry in patients with stroke (14). Music-based interventions have also been associated with improvements in lower limb motor function and balance (15). In upper limb rehabilitation, music-supported therapy has been shown to enhance the flexibility and coordination of the affected hand (16). In addition, music-based interventions may help alleviate anxiety and depression and improve adherence to rehabilitation exercises (17). In terms of quality of life, music-based interventions have also been found to improve stroke survivors’ emotional state, social communication, and overall perception of health (18). Notably, even in the late post-stroke period, multimodal rehabilitation interventions can still improve patients’ grip strength, balance, and self-perceived recovery (19).

The physiological mechanisms of auditory-motor coupling provide theoretical support for the efficacy of music-based interventions (20). However, the current evidence regarding the effectiveness of music-based interventions in stroke rehabilitation remains inconclusive. There are significant variations across studies in intervention protocols, duration of intervention, and outcome measures. Furthermore, most studies have small sample sizes, lack long-term follow-up, and have insufficient control of bias risks. Therefore, it is necessary to integrate the evidence from existing randomized controlled trials through systematic reviews and meta-analyzes to more comprehensively evaluate the rehabilitation effects of music-based interventions for stroke patients.

## 2 Methods

This study was registered in PROSPERO (CRD420261419159) and conducted in accordance with the PRISMA 2020 statement (21).

### 2.1 Literature search strategy

A systematic search was conducted in Web of Science, PubMed, Embase, Cochrane Library, Scopus, ProQuest, CINAHL, SinoMed (CBM), China National Knowledge Infrastructure (CNKI), Wanfang Database, and Technology Journal Database (VIP), covering all records from their inception through April 7, 2026. The search strategy combined subject headings and free-text terms related to stroke and music-based interventions. The detailed search strategy for each database is provided in Supplementary Table S1.

### 2.2 Inclusion and exclusion criteria

Following the PICOS principles (participants, interventions, control, outcomes, and study design), the studies that matched the following criteria were included: (1) P: Stroke patients aged ≥18 years; (2) I: Intervention groups received music-based interventions in which music or rhythmic auditory cues constituted the primary difference from the control intervention; (3) C: Control groups received comparable interventions without musical components, with the musical component being the principal difference between the intervention and control groups; (4) O: Outcome indicators include upper limb function, gait and balance, activities of daily living, and quality of life; (5) S: Exclusively encompassing randomized controlled trials.

The exclusion criteria: (1) Unable to access full text; (2) Sample size ≤15; (3) The intervention measures in the intervention and control groups were unclear, or the two groups were not comparable; (4) No providing sufficient data for further analysis; (5) Stroke rehabilitation-related outcome measures were not reported.

### 2.3 Data extraction

Data were independently extracted from the included studies by two reviewers using a standardized data extraction form. The extracted information included general study information (first author and publication year), participants’ characteristics (sample size and age), the brief details of the intervention and the control groups, the intervention duration and outcome measures. Any disagreements were resolved through discussion or consultation with a third reviewer.

### 2.4 Risk of bias and methodological quality assessment

The risk of bias in the included studies was independently assessed by two reviewers using the Cochrane Risk of Bias tool described in the Cochrane Handbook for Systematic Reviews of Interventions (version 5.1.0) (22). Assessment items include: (1) random sequence generation; (2) allocation concealment (selection bias); (3) blinding of investigators and participants (performance bias); (4) blinding of outcome assessment (detection bias); (5) completeness of data (attrition bias); (6) selective reporting (reporting bias); (7) other bias. Each domain was rated as low, high, or unclear risk of bias. Any disagreements were resolved through discussion or consultation with a third reviewer.

In addition, the methodological quality of the included randomized controlled trials was evaluated using the Physiotherapy Evidence Database (PEDro) scale. The PEDro scale consists of 11 items, of which 10 contribute to the total score (range: 0–10), with higher scores indicating better methodological quality. Two reviewers independently performed the PEDro assessment, and disagreements were resolved through discussion or consultation with a third reviewer. The Cochrane Risk of Bias tool was used to assess domain-specific risks of bias, whereas the PEDro scale was applied to provide an overall evaluation of methodological quality. Item-level PEDro scores are presented in Supplementary Table S4.

### 2.5 Quality of evidence assessment

The certainty of the evidence was assessed using the Grading of Recommendations Assessment, Development and Evaluation (GRADE) framework (23). Evidence from randomized controlled trials was initially rated as high quality and was subsequently downgraded, when appropriate, according to five domains: risk of bias, inconsistency, indirectness, imprecision, and publication bias. Finally, the final GRADE evidence quality level (High/Moderate/Low/Very Low) was determined through a comprehensive evaluation.

### 2.6 Statistical analyzes

The meta-analysis was performed using Review Manager (RevMan) version 5.4. The required data, including means, standard deviations (SDs), and sample sizes, were extracted and entered into RevMan for statistical analysis. If the outcome was measured using the same instrument, the mean difference (MD) was used for effect value selection for continuous variables. If the outcome indicators were measured using a different instrument, the standardized mean difference (SMD) as well as the 95% CIs were selected as the effect value. Statistical heterogeneity was assessed using Cochran’s *Q* test and the *I*^2^ statistic. A random-effects model was used when substantial heterogeneity was detected (*p* < 0.10 and *I*^2^ ≥ 50%); otherwise, a fixed-effects model was applied. Subgroup analysis should be conducted to explore the causes of heterogeneity. *p* < 0.05 was considered a statistically significant difference. Sensitivity analyzes were conducted by sequentially excluding individual studies to evaluate the robustness of the pooled results. Because fewer than 10 studies were included for each outcome, publication bias was assessed qualitatively using funnel plots.

## 3 Results

### 3.1 Literature search

**Figure 1:**
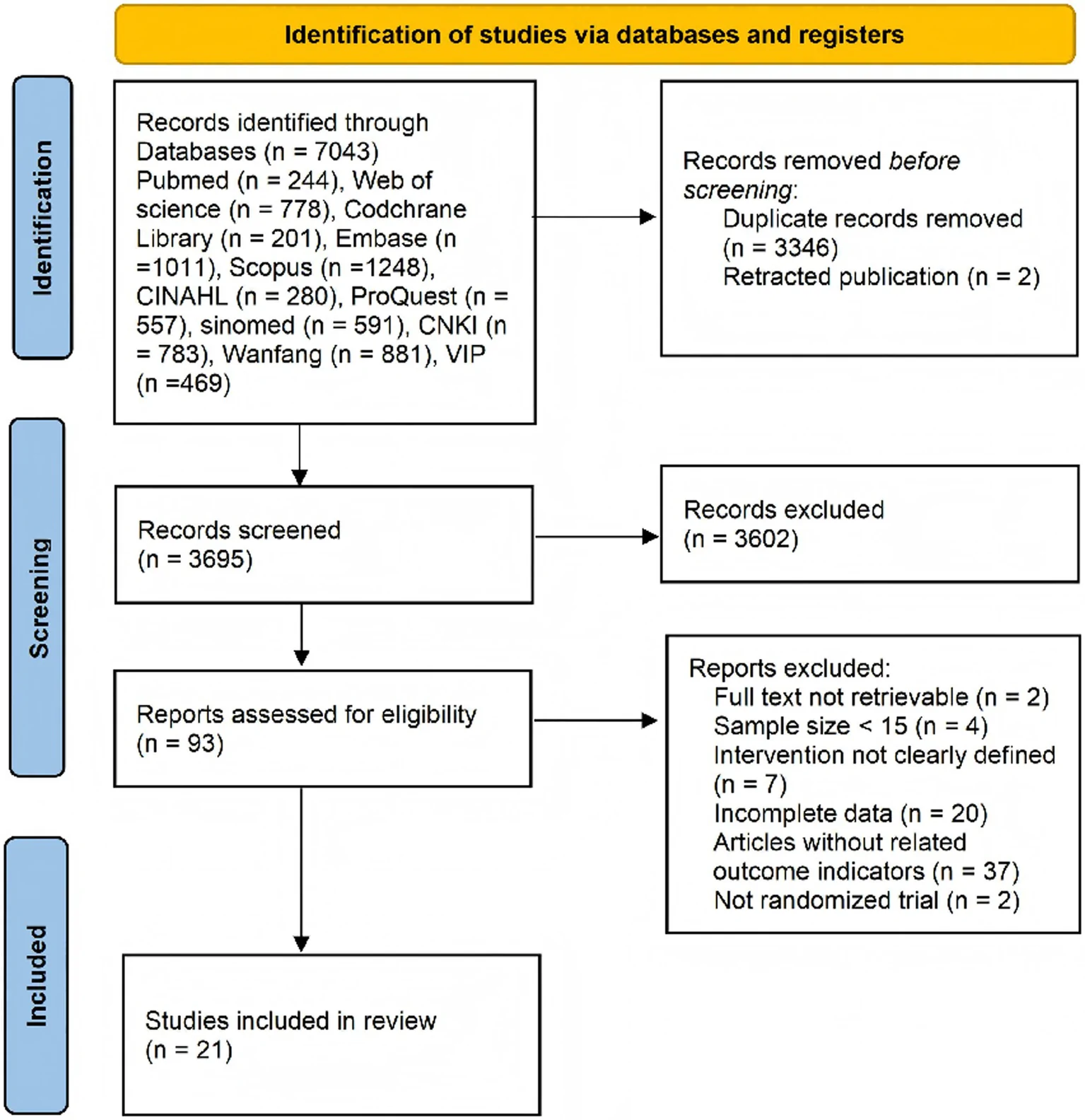
Flow diagram of the studies included in the meta-analysis.

A total of 7,043 records were identified through searches of 11 electronic databases. Two retracted publications were removed. After removing 3,346 duplicate records using EndNote 2025 and manual checking, 3,695 records remained for title and abstract screening. Following screening, we evaluated the full text of 93 studies. Among these, studies that were not randomized controlled trials, had a sample size of fewer than 15 participants, lacked a clear intervention, had incomplete data, lacked relevant outcome measures and were unable to obtain full text were excluded. Finally, 21 studies met the inclusion criteria and were included in the meta-analysis (2, 3, 7, 9, 10–20, 24–29). The study selection process is presented in Figure 1.

### 3.2 Characteristics of the included studies

A total of 21 randomized controlled trials (RCTs) were included in this review. The studies were published between 1997 and 2024 and involved 1,029 participants (intervention: 519; control: 510). The intervention group received music-based interventions such as rhythmic auditory stimulation, music-supported therapy, and music-based movement training, while the control group received conventional rehabilitation training or standard care. The intervention duration ranged from 3 to 12 weeks, with 4 weeks being the most common. Primary outcome measures included upper limb motor function, gait and balance, activities of daily living, and quality of life. The basic characteristics of the included studies are shown in Table 1. Details of time since stroke, stroke stage, baseline cognitive assessment, and cognitive eligibility criteria are presented in Supplementary Table S2. Most studies included patients in the subacute or chronic stage. Baseline cognitive status was not reported in several studies. Among those that reported cognitive eligibility, participants were generally required to have a minimum MMSE score or no severe cognitive impairment.

**Table 1:** Characteristics of selected 21 studies included in the systematic review.

Author, year	Sample (E/C)	Mean age	Interventions		Duration	Outcomes
			EG	CG		
Thaut 1997 (14)	10/10	EG: 73.00 ± 7.00 CG: 72.00 ± 8.00	RAS gait training + physical therapy	physical therapy	6 weeks	Gait and balance
Schauer 2003 (24)	11/12	EG: 59.00 ± 12.00 CG: 61.00 ± 12.00	MMF gait training + NDT	Conventional gait training + NDT	3 weeks	Gait and balance
Thaut 2007 (16)	43/35	EG: 69.20 ± 11.50 CG: 69.70 ± 11.20	RAS gait training	NDT/Bobath gait training	3 weeks	Gait and balance
Cha 2014 (10)	10/10	EG: 59.80 ± 11.70 CG: 63.00 ± 14.10	RAS gait training	Intensive gait training	6 weeks	Gait and balance Quality of life
Suh 2014 (25)	8/8	EG: 61.00 ± 14.48 CG:70.63 ± 12.42	RAS gait training + NDT	Gait training + NDT	3 weeks	Gait and balance
Tong 2015 (11)	15/15	EG: 50.10 ± 14.80 CG:48.60 ± 14.60	Audible musical-instrument training + conventional rehabilitation	Mute musical-instrument training + conventional rehabilitation	4 weeks	Upper-limb motor function
Song 2016 (3)	20/20	EG: 57.10 ± 7.80 CG: 60.10 ± 6.80	RAS gait training + NDT	Supervised gait training + NDT	4 weeks	Gait and balance
Pocwierz-Marciniak 2017 (27)	30/31	EG: 65.00 CG: 63.00	Individual music therapy + standard care	Standard care	5 weeks	Quality of life
Jia 2017 (26)	25/25	EG: 58.00 ± 12.00 CG: 61.00 ± 11.00	Music therapy + routine rehabilitation	Routine rehabilitation	4 weeks	Upper-limb motor function ADL
Bunketorp-Käll 2017 (19)	40/41	EG: 62.70 ± 6.70 CG: 63.70 ± 6.70	Rhythm‑and‑music therapy(R-MT)	Routine	12 weeks	Gait and balance Quality of life
Raglio 2017 (9)	19/19	EG: 70.40 ± 8.90 CG:75.40 ± 7.60	Active music therapy + standard rehabilitation	Standard rehabilitation	6–8 weeks	Gait and balance ADL Quality of life
Jia 2018 (8)	20/20	EG: 63.15 ± 8.56 CG: 62.60 ± 9.25	Individual music therapy + conventional rehabilitation	Conventional rehabilitation	4 weeks	Upper-limb motor function Gait and balance
Zhou 2018 (17)	30/30	EG: 63.42 ± 12.46 CG: 62.23 ± 13.26	Musical rhythm passive-limb exercise + routine	Routine care	8 weeks	ADL
Fujioka 2018 (18)	14/14	EG: 64.20 ± 9.40 CG: 54.30 ± 11.30	Music-supported upper-limb training	Repetitive upper-limb exercise	10 weeks	Quality of life
Grau-Sánchez 2018 (20)	19/20	EG: 60.10 CG: 62.50	MST + standard rehabilitation	Standard rehabilitation	4 weeks	Upper-limb motor function Quality of life
Hu 2021 (28)	29/29	EG: 73.00 ± 8.00 CG: 72.30 ± 7.50	Music therapy + conventional rehabilitation	Conventional rehabilitation	4 weeks	Upper-limb motor function Gait and balance ADL
Wang 2021 (15)	75/75	EG: 59.87 ± 11.48 CG: 60.23 ± 11.79	Music-based exercise therapy + traditional rehabilitation	Conventional exercise therapy + traditional rehabilitation	4 weeks	Upper-limb motor function Gait and balance ADL Quality of life
Raglio A 2021 (12)	33/32	EG: 62.40 ± 8.90 CG: 64.70 ± 16.00	Sonification-assisted upper-limb training + usual care	Standard upper-limb training + usual care	4 weeks	Upper-limb motor function Quality of life
Wang Y 2021 (15)	30/30	EG: 61.12 ± 7.49 CG: 61.02 ± 7.51	Rhythmic music therapy + routine gait training + conventional treatment	Routine gait training + conventional treatment	4 weeks	Gait and balance
Ding 2023 (29)	19/19	EG: 65.72 ± 15.18 CG: 71.47 ± 10.14	Music-assisted task-oriented training + conventional occupational therapy	Conventional occupational therapy	4 weeks	Upper-limb motor function ADL
Fan 2024 (13)	18/16	EG: 58.00 ± 5.33 CG: 60.88 ± 5.82	Personalized music listening + standard routine care	Standard routine care	3 months	ADL

EG, experimental group; CG, control group; RAS, Rhythmic Auditory Stimulation; MMF, Musical Motor Feedback; MST, Music-Supported Therapy; R-MT, Rhythm-and-Music Therapy; ADL, Activities of Daily Living.

### 3.3 Risk of bias and methodological quality assessment

**Figure 2:**
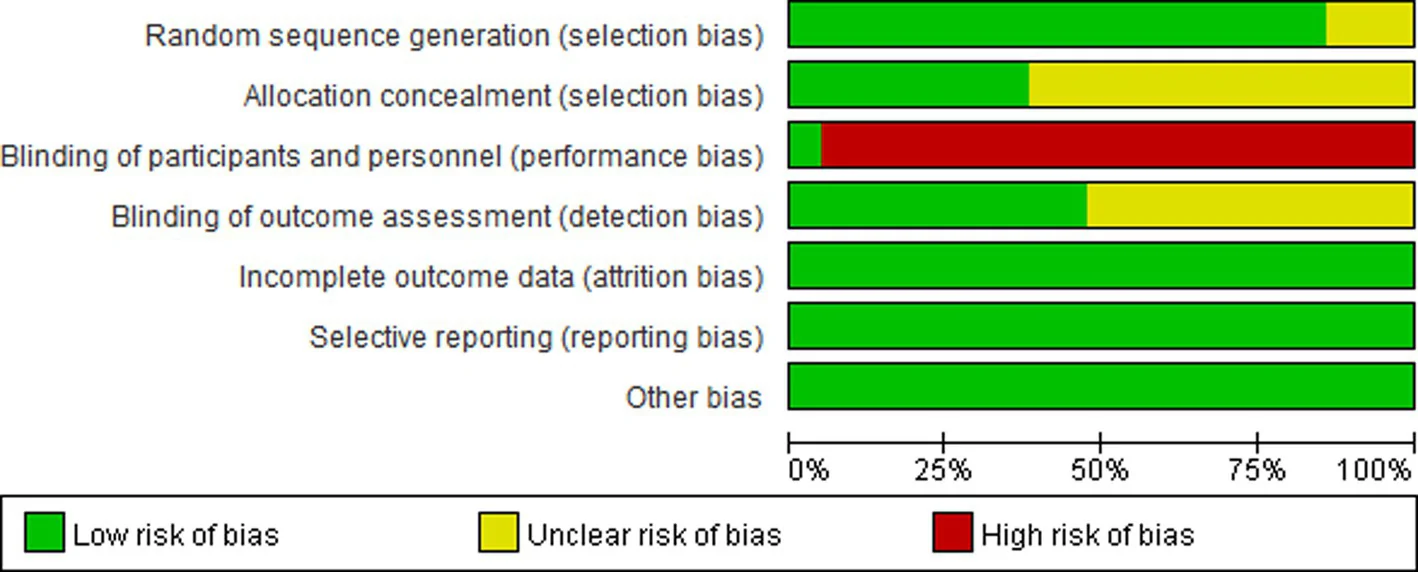
Summary of the quality evaluation and risk of bias.

**Figure 3:**
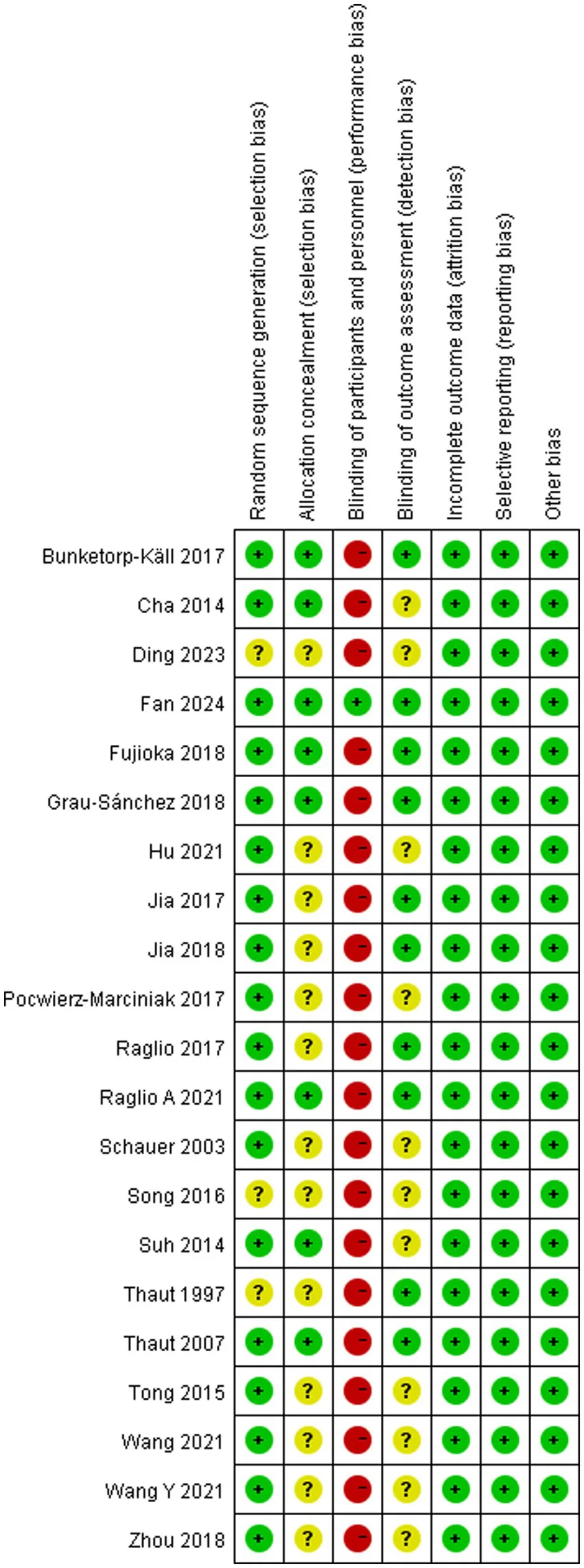
Risk of bias analysis of each included study.

An overview of the risk-of-bias assessment is presented in Figure 2. Most of the included studies were judged to have a low risk of bias in terms of incomplete outcome data, selective reporting, and other sources of bias. Due to the nature of the interventions, blinding of participants and personnel was not feasible in most studies, resulting in a high risk of performance bias. In addition, several studies did not provide sufficient information regarding allocation concealment or blinding of outcome assessment and were therefore rated as having an unclear risk of bias in these domains. Detailed risk-of-bias judgments for each included study are presented in Figure 3.

PEDro scores ranged from 5 to 8. Most studies were rated as good methodological quality, whereas a few were rated as acceptable quality. No studies were classified as low quality according to the PEDro scale. Detailed PEDro item scores are presented in Supplementary Table S4.

### 3.4 Quality of evidence

According to the GRADE assessment, the certainty of the evidence was rated as moderate for upper limb motor function, gait speed, balance, activities of daily living, and quality of life, whereas the certainty of the evidence for lower limb motor function was rated as low. All outcomes were downgraded due to risk of bias, and lower limb motor function was further downgraded because of substantial heterogeneity. No serious concerns regarding indirectness, imprecision, or publication bias were identified. Detailed GRADE assessments are presented in Table 2.

**Table 2:** GRADE evidence quality for meta-analysis of RCTs.

Outcome	*n*	Risk of bias	Inconsistency	Indirectness	Imprecision	Publication bias	GRADE
Upper limb motor function	8	Serious	Not serious (*I*^2^ = 63%, explainable heterogeneity)	Not serious	No serious	Undetected	⊕ ⊕ ⊕ ○ (Moderate)
Gait speed	7	Serious	Not serious (*I*^2^ = 79%, explainable heterogeneity)	Not serious	Not serious	Undetected	⊕ ⊕ ⊕ ○ (Moderate)
Balance function	4	Serious	Not serious (*I*^2^ = 41%)	Not serious	Not serious	Undetected	⊕ ⊕ ⊕ ○ (Moderate)
Lower limb motor function	4	Serious	Serious (*I*^2^ = 59%)	Not serious	Not serious	Undetected	⊕ ⊕ ○ ○ (Low)
Activities of daily living	7	Serious	Not serious (*I*^2^ = 7%)	Not serious	Not serious	Undetected	⊕ ⊕ ⊕ ○ (Moderate)
Quality of life	8	Serious	Not serious (*I*^2^ = 19%)	Not serious	Not serious	Undetected	⊕ ⊕ ⊕ ○ (Moderate)

### 3.5 Meta-analysis results

#### 3.5.1 Upper limb motor function

**Figure 4:**
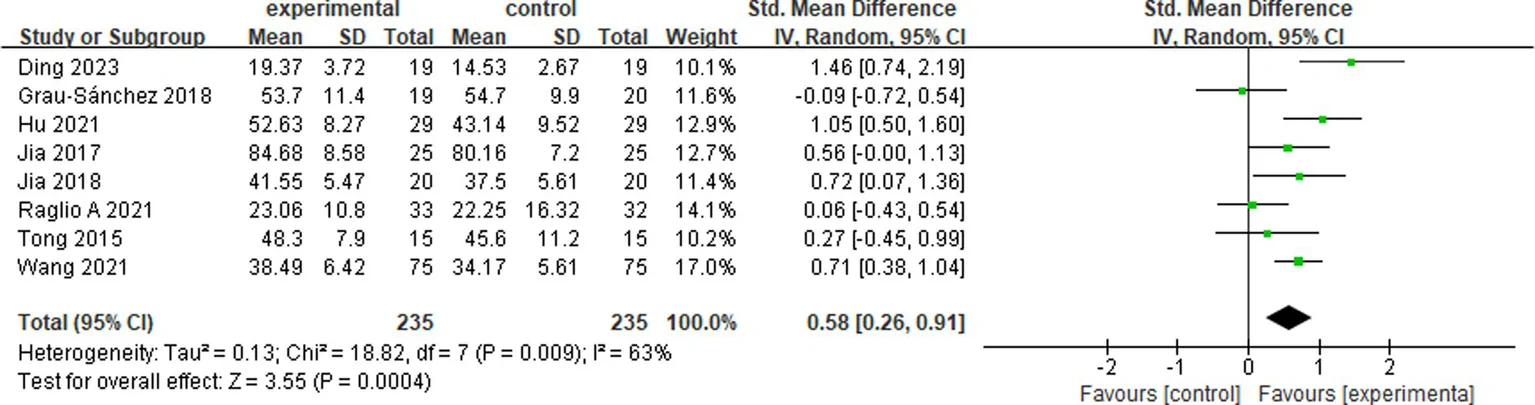
Forest plot of the effects of music-based interventions on upper limb motor function after the intervention.

Eight studies (2, 7, 11, 12, 20, 26, 28, 29) were included in the meta-analysis to evaluate the effects of music-based interventions on upper limb motor function in stroke patients. A total of 470 participants were enrolled, with 235 participants in the intervention group and 235 in the control group. The assessment scales included the Fugl-Meyer Assessment (FMA) and the Box and Block Test (BBT). As the upper limb motor function assessment scales used varied across studies, the standardized mean difference (SMD) was employed as the effect measure. The heterogeneity analysis indicated moderate heterogeneity among the included studies (*I*^2^ = 63%, *p* = 0.009). Therefore, a random-effects model was applied. The meta-analysis results showed that, compared with the control group, music-based interventions significantly improved upper limb motor function in stroke patients, with the difference being statistically significant [SMD = 0.58, 95% CI (0.26, 0.91), *p* = 0.0004] (Figure 4).

#### 3.5.2 Gait and balance

**Figure 5:**
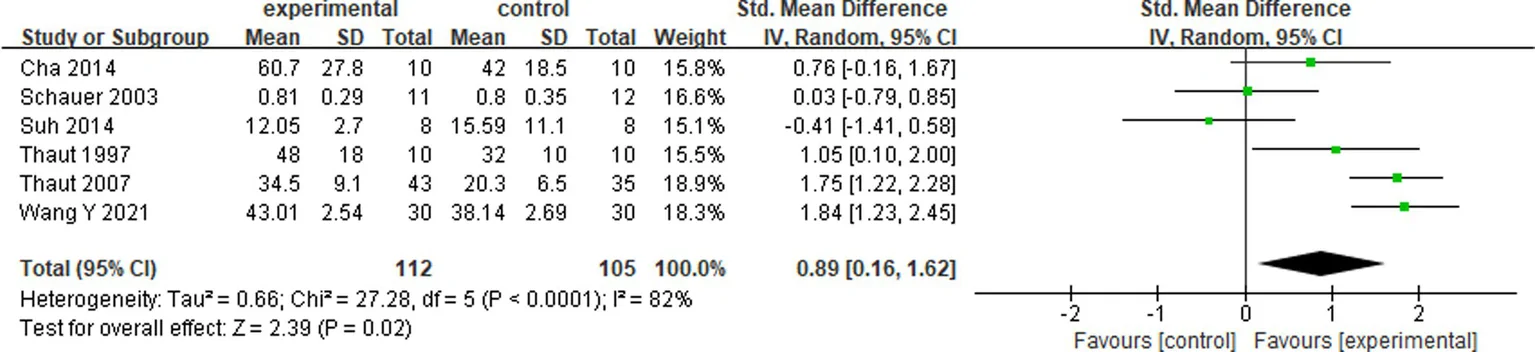
Forest plot of the effects of music-based interventions on gait speed after the intervention.

This study evaluated the effects of music-based interventions on gait speed in patients with stroke. Seven studies (3, 10, 14, 15, 16, 24, 25) reported gait-related outcomes. Among them, six studies (10, 14, 15, 16, 24, 25) assessed gait speed, whereas one study (3) assessed walking time using the 10-m Walk Test. Although gait speed was reported in different units (m/s, cm/s, and m/min), all studies evaluated the same outcome, and higher values consistently indicated better gait performance. Therefore, the standardized mean difference (SMD) was used to pool the results. Substantial heterogeneity was observed among the included studies (*I*^2^ = 82%, *p* < 0.001), and a random-effects model was applied. Music-based interventions significantly improved gait speed compared with the control group [SMD = 0.89, 95% CI (0.16, 1.62), *p* = 0.02] (Figure 5). One study reported walking time using the 10-m Walk Test. Because this outcome was reported in only one study and differed conceptually from gait speed, it was summarized descriptively rather than pooled. The post-intervention walking time was 16.7 ± 8.9 s in the intervention group and 14.3 ± 9.3 s in the control group. The intervention group had a longer walking time at baseline but demonstrated greater improvement after the intervention.

**Figure 6:**
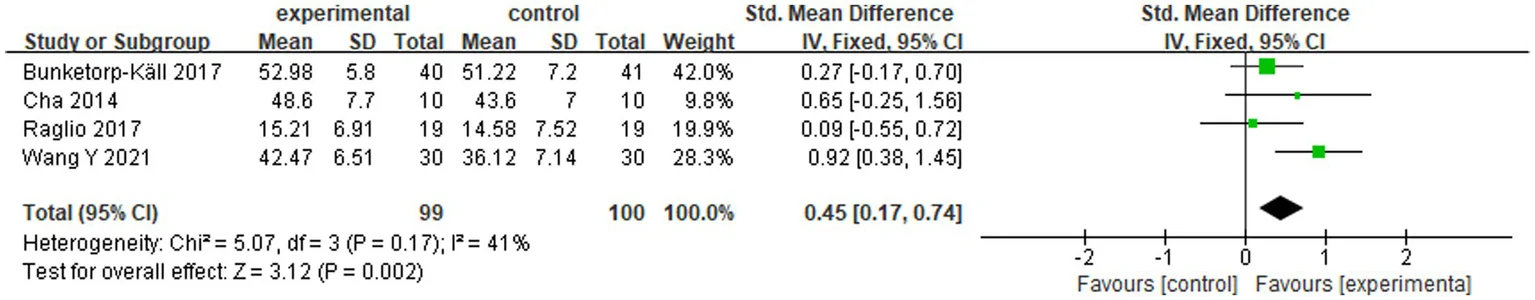
Forest plot of the effects of music-based interventions on balance function after the intervention.

For balance function, four studies (9, 10, 15, 19) were included in the meta-analysis, with balance outcomes assessed using the Berg Balance Scale (BBS) and other validated measures. The heterogeneity analysis showed low between-study heterogeneity (*I*^2^ = 41%, *p* = 0.17); therefore, a fixed-effects model was applied. The meta-analysis demonstrated that music-based interventions significantly improved balance function in stroke patients compared with the control group, with a statistically significant difference between groups [SMD = 0.45, 95% CI (0.17, 0.74), *p* = 0.002] (Figure 6).

**Figure 7:**
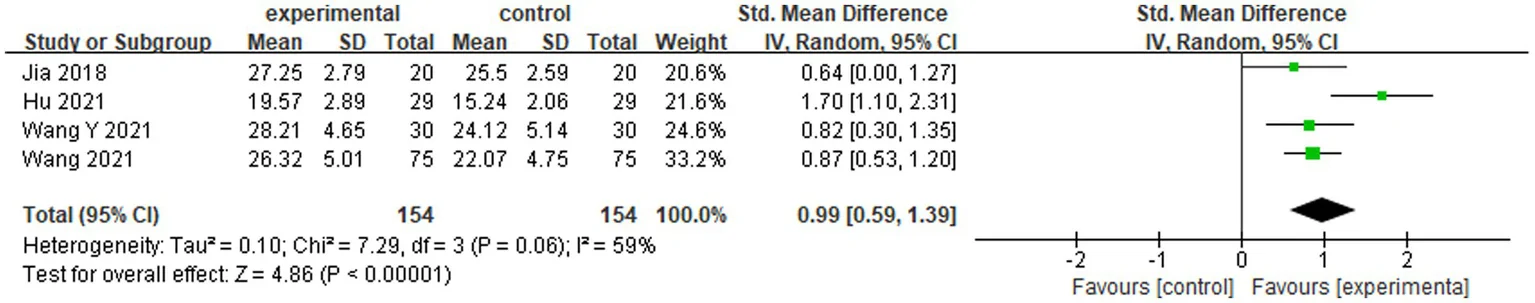
Forest plot of the effects of music-based interventions on lower limb motor function after the intervention.

In addition, the effects of music-based interventions on lower limb motor function were evaluated. Four studies (2, 7, 15, 28) were included in the meta-analysis, which assessed lower limb motor function in stroke patients using scales such as the Fugl-Meyer Assessment-Lower Extremity (FMA-LE). Heterogeneity analysis indicated moderate heterogeneity among the included studies (*I*^2^ = 59%, *p* = 0.06); therefore, a random-effects model was used. The meta-analysis results indicated that, compared with the control group, music-based interventions improved lower limb motor function in stroke patients [SMD = 0.99, 95% CI (0.59, 1.39), *p* < 0.001] (Figure 7).

#### 3.5.3 Activities of daily living (ADL)

**Figure 8:**
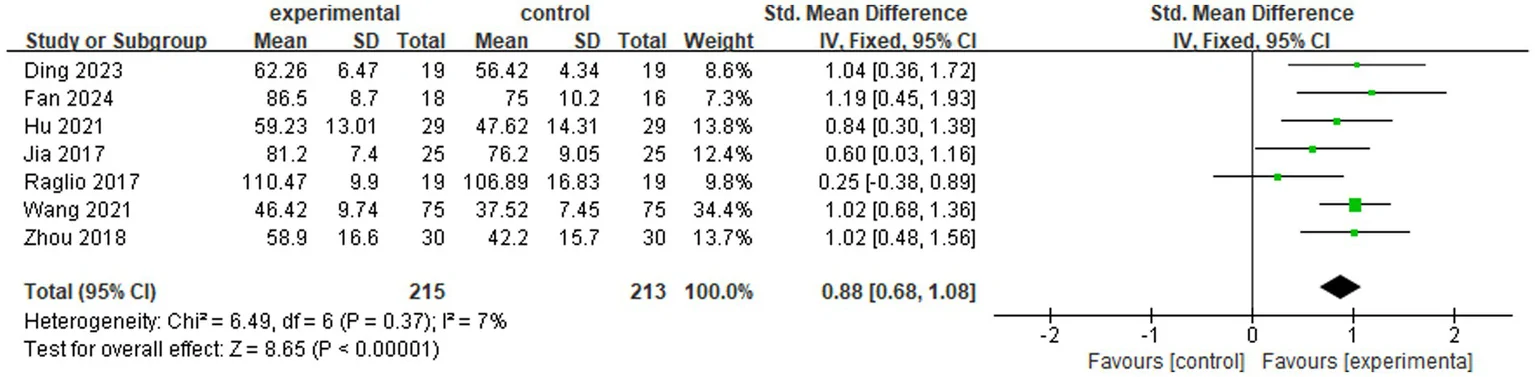
Forest plot of the effects of music-based interventions on activities of daily living after the intervention.

Seven studies (2, 9, 13, 17, 26, 28, 29) involving 428 participants were included in the meta-analysis to evaluate the effects of music-based interventions on activities of daily living in stroke patients. As different assessment tools were used across studies, including the Barthel Index (BI) and the Functional Independence Measure (FIM), the standardized mean difference (SMD) was used as the pooled effect size. The heterogeneity analysis indicated low heterogeneity among the included studies (*I*^2^ = 7%, *p* = 0.37); therefore, a fixed-effects model was applied. The results of the meta-analysis showed that music-based interventions significantly improved activities of daily living in stroke patients compared with control interventions, and the difference was statistically significant [SMD = 0.88, 95% CI (0.68, 1.08), *p* < 0.001] (Figure 8).

#### 3.5.4 Quality of life (QoL)

**Figure 9:**
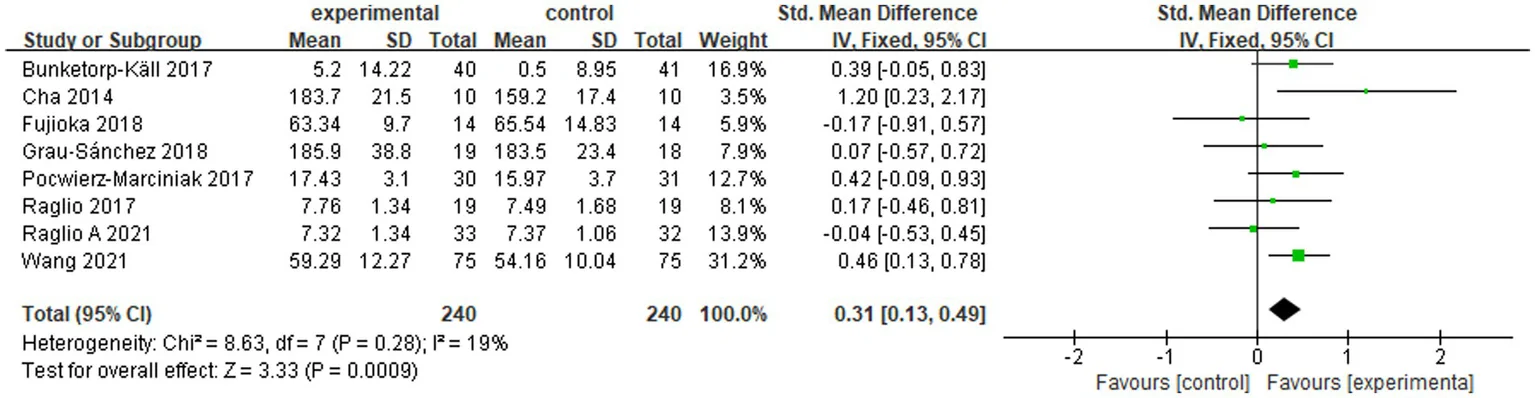
Forest plot of the effects of music-based interventions on quality of life after the intervention.

Eight studies (2, 9, 10, 12, 18, 19, 20, 27) involving 480 patients with stroke were included in the meta-analysis to assess the effects of music-based interventions on quality of life. As different quality-of-life assessment instruments were used across the included studies, the standardized mean difference (SMD) was selected as the pooled effect size. The heterogeneity analysis showed low heterogeneity among studies (*I*^2^ = 19%, *p* = 0.28); therefore, a fixed-effects model was applied. The results of the meta-analysis showed that music-based interventions significantly improved quality of life in stroke patients compared with the control group, with a statistically significant between-group difference [SMD = 0.31, 95% CI (0.13, 0.49), *p* = 0.0009] (Figure 9).

### 3.6 Subgroup analysis

#### 3.6.1 Upper limb motor function

Subgroup analysis was performed according to the different types of music-based interventions in the intervention groups. The included studies were classified into active, receptive music, and mixed music interventions.

**Figure 10:**
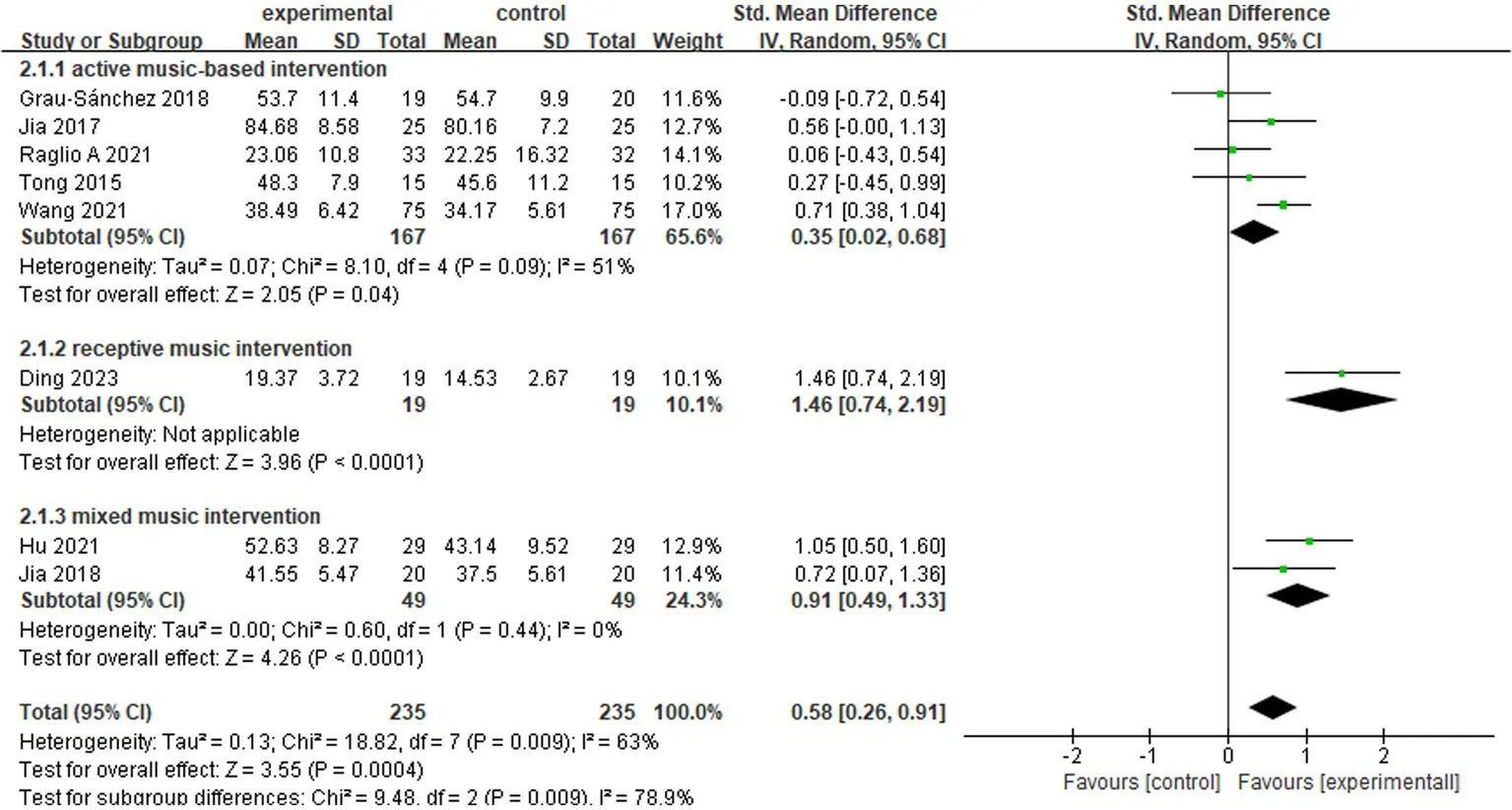
Forest plot of the subgroup analysis of the effects of music-based interventions on upper limb motor function according to intervention type.

The findings showed that heterogeneity was reduced in both the active music intervention subgroup (*I*^2^ = 51%, *p* = 0.09) and the mixed music intervention subgroup (*I*^2^ = 0%, *p* = 0.44). Among these, five studies (2, 11, 12, 20, 26) were included in the active music intervention subgroup. The pooled results indicated that active music-based interventions significantly improved upper limb motor function in stroke patients compared with the control group [SMD = 0.35, 95% CI (0.02, 0.68), *p* = 0.04], with moderate heterogeneity observed within the subgroup (*I*^2^ = 51%). The receptive music intervention subgroup included only one study (29), which showed a significant benefit in favor of the intervention group [SMD = 1.46, 95% CI (0.74, 2.19), *p* < 0.001]. Two studies (7, 28) were included in the mixed music intervention subgroup. The pooled analysis demonstrated a significant improvement in upper limb motor function among patients receiving mixed music interventions [SMD = 0.91, 95% CI (0.49, 1.33), *p* < 0.001], with no significant heterogeneity observed within the group (*I*^2^ = 0%). The test for subgroup differences was statistically significant (*p* = 0.009), indicating that the type of music intervention may have contributed to the heterogeneity observed across studies (Figure 10).

#### 3.6.2 Gait and balance

**Figure 11:**
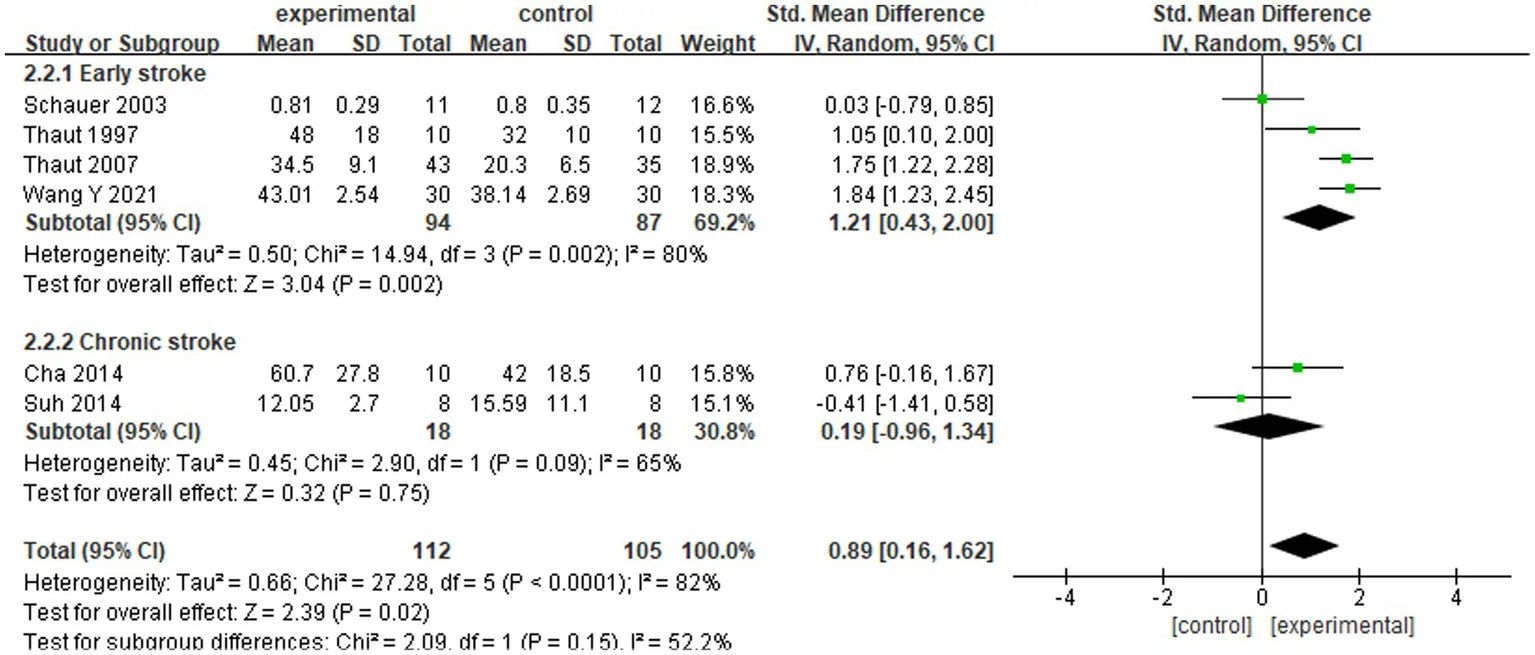
Forest plot of the subgroup analysis of the effects of music-based interventions on gait speed according to stroke stage.

To explore the influence of stroke stage, the included studies were grouped into early-stage and chronic stroke subgroups. Music-based interventions significantly improved gait speed in patients with early-stage stroke [SMD = 1.21, 95% CI (0.43, 2.00), *p* = 0.002], whereas no significant improvement was observed in patients with chronic stroke [SMD = 0.19, 95% CI (−0.96, 1.34), *p* = 0.75]. However, the subgroup difference was not statistically significant (*p* = 0.15). High heterogeneity persisted in both subgroups (Figure 11).

A leave-one-out sensitivity analysis was also conducted. Sequential omission of individual studies did not meaningfully alter the pooled results, and heterogeneity remained substantial throughout the analyzes. This suggests that the observed heterogeneity was not attributable to any single study.

### 3.7 Publication bias

As fewer than 10 studies were included for each outcome, publication bias was assessed qualitatively using funnel plots only. For upper limb motor function (*n* = 8), the funnel plot showed an approximately symmetrical distribution of studies (Supplementary Figure S1), suggesting no obvious evidence of publication bias. Similarly, the funnel plot for gait speed (*n* = 6) appeared generally symmetrical (Supplementary Figure S2), indicating no apparent publication bias. For the balance function (*n* = 4) and lower limb motor function (*n* = 4), the small number of included studies resulted in greater dispersion within the funnel plots (Supplementary Figures S3, S4). Therefore, these findings should be interpreted with caution. Funnel plots for activities of daily living (*n* = 7) and quality of life (*n* = 8) also showed an approximately symmetrical distribution of studies (Supplementary Figures S5, S6), suggesting no obvious publication bias. However, due to the limited number of included studies, the results should be interpreted with caution.

### 3.8 Sensitivity analysis

A sensitivity analysis was conducted using a leave-one-out approach to evaluate the influence of individual studies on the overall findings. The results showed that the pooled effect size remained largely unchanged after the removal of any single study, and the overall conclusions were consistent across analyzes. These findings indicate that the results of this meta-analysis are robust and stable, with no individual study exerting a substantial influence on the overall effect estimate.

## 4 Discussion

This meta-analysis included 21 randomized controlled trials involving 1,029 participants. The findings demonstrated that compared with conventional rehabilitation, music-based interventions significantly improved upper limb motor function, gait and balance, activities of daily living, and quality of life. As a non-pharmacological adjunct to conventional rehabilitation, music-based interventions may help improve functional independence and support the overall rehabilitation process after stroke (30, 31).

Post-stroke functional impairments commonly affect multiple domains, including motor function, gait and postural control, activities of daily living, cognition, and emotional wellbeing. As a result, the rehabilitation process is often complex and prolonged (32). In recent years, the process of rehabilitation practice has increasingly emphasized patients’ active participation and the application of various sensory stimuli in training (33). The music-based interventions included in this review were diverse and included rhythmic auditory stimulation, music-supported therapy, instrument-based training, and music listening (30). Although these approaches differ in their implementation, they all integrate musical or auditory stimuli into rehabilitation activities. Rhythmic auditory stimulation is commonly applied to gait training by providing external temporal cues for movement (14), whereas music-supported therapy and instrument-based training are more frequently used to improve upper limb motor function (34). Because these interventions involve responding to auditory cues during movement training, patients’ cognitive function may influence their participation in rehabilitation and the effectiveness of treatment. In addition, receptive music therapy may enhance emotional engagement and motivation during rehabilitation (35). Such differences in intervention characteristics may partly explain the heterogeneity observed across studies. Nevertheless, all approaches share the common goal of increasing patient participation and engagement in rehabilitation, which may contribute to the overall positive effects of music-based interventions on functional recovery after stroke (36, 37).

With regard to upper limb function, the results of the meta-analysis indicate that music-based interventions are more effective than conventional rehabilitation in improving outcomes. The subgroup analysis suggested possible differences among the intervention categories, with mixed interventions showing a larger pooled effect than the other subgroups. One possible explanation is that active music therapy emphasize patients’ active involvement in rehabilitation activities, while receptive music therapy continuously provides auditory and emotional stimulation. The combination of these approaches may not only enhance participation in training but also maximize the therapeutic effects of musical stimulation (38). However, only a limited number of studies were included in each subgroup, so these findings should be interpreted with caution. The observed differences may also be influenced by variations in participant characteristics, intervention protocols, and study quality. Therefore, these subgroup results should be regarded as exploratory, and further well-designed studies are needed to confirm these findings.

Music-based interventions significantly improved gait speed in patients with stroke. This finding is consistent with previous systematic reviews reporting beneficial effects of music-based interventions on gait recovery after stroke (39, 40). Gait speed is widely recognized as an important indicator of functional recovery after stroke and is closely associated with independence in daily activities and community mobility (41). Although gait speed was reported using different units across studies, all studies evaluated the same outcome, making pooled analysis using standardized mean differences appropriate. Considerable heterogeneity was observed in the pooled analysis. Neither subgroup analysis by stroke stage nor leave-one-out sensitivity analysis substantially reduced the heterogeneity, suggesting that it was unlikely to be explained by a single factor. Variations in intervention protocols, rehabilitation intensity, baseline functional status, and outcome assessment may all have contributed to the observed differences across studies. Music-based interventions also improved balance and lower limb motor function. Both balance ability and lower limb function are fundamental components of walking recovery (42). Although the effect size for balance function was relatively modest, the low level of heterogeneity suggests good consistency across the included studies. The improvement in lower limb motor function further supports the potential value of music-based interventions in promoting the recovery of walking-related functions. These findings indicate that music-based interventions may facilitate gait recovery by improving multiple aspects of motor function. However, the underlying mechanisms remain unclear and warrant further investigation.

The results of the meta-analysis indicate that music-based interventions can improve the ability of stroke patients to perform activities of daily living (ADL). The ability to perform activities of daily living is an important indicator for assessing the overall level of functional recovery in stroke patients (43). Improvements in this area mean that patients are better able to carry out basic activities such as eating, dressing and transferring (44). As discussed previously, music-based interventions have been shown to facilitate improvements in upper limb motor function, gait and balance, and lower limb motor function, all of which are important for ADL performance (45). Therefore, the observed benefits in ADL may result from the combined effects of recovery across multiple functional domains. In addition, low heterogeneity was identified for this outcome, indicating good consistency among the included studies and further supporting the reliability of these findings.

With regard to quality of life, music-based interventions can further improve the quality of life of stroke patients beyond that achieved through conventional rehabilitation. Although the overall effect size was relatively modest, the results were statistically significant and there was low heterogeneity between studies, suggesting that this beneficial effect is fairly consistent. Quality of life is a comprehensive outcome that reflects an individual’s overall health status and is influenced by multiple factors, including physical function, emotional wellbeing, social participation, and the ability to perform daily activities (46). This review also found that music-based interventions have a positive impact on motor function and the ability to perform activities of daily living. Therefore, the improvement in quality of life may be the result of the combined effects of functional recovery across multiple domains. Furthermore, as a non-pharmacological intervention, music-based interventions have the potential to improve emotional wellbeing and alleviate negative psychological symptoms such as depression and anxiety, thereby contributing to improved quality of life (47).

Overall, the findings of this review suggest that music-based interventions may serve as a valuable adjunct to conventional stroke rehabilitation. By integrating musical elements into therapeutic activities, music-based interventions appear to support recovery across multiple functional domains while also facilitating patient engagement in rehabilitation. Given their low cost, safety, and ease of implementation, music-based interventions may represent a promising strategy for improving rehabilitation outcomes in stroke patients.

## 5 Limitations

This study has the following limitations. First, only published articles in Chinese and English were included, which may have limited the comprehensiveness of the available evidence. Future reviews should consider including studies published in other languages to reduce the potential risk of language bias. Second, although all included studies were randomized controlled trials, some were insufficiently reporting on methodological aspects such as blinding and allocation concealment, which may have affected the reliability of the results. Third, considerable variation was observed in the type of music-based interventions, intervention frequency, and treatment duration across studies, which may have contributed to the clinical heterogeneity of the findings. In addition, most of the included studies did not report participants’ baseline cognitive function, making it difficult to determine whether cognitive status influenced the effectiveness of music-based interventions. Some interventions, particularly rhythmic auditory stimulation and music-supported therapy, require patients to recognize auditory cues and respond with coordinated movements. Therefore, the findings should be interpreted with caution in patients with severe cognitive impairment. Future studies should include baseline cognitive assessment and further explore its influence on rehabilitation outcomes. Finally, most studies focused on short-term outcomes assessed immediately after the intervention, whereas long-term follow-up data were limited. Therefore, further research is needed to determine whether the benefits of music-based interventions on functional recovery and quality of life can be maintained over time in individuals with stroke.

## 6 Conclusion

In conclusion, the current evidence suggests that music-based interventions may improve upper limb motor function, gait and balance, activities of daily living, and quality of life in stroke patients. These findings support the potential value of incorporating music-based interventions into stroke rehabilitation. Further randomized controlled trials with more rigorous designs, larger sample sizes and longer follow-up periods are needed to determine the optimal intervention protocols and provide stronger evidence for clinical practice.

## Data Availability

The original contributions presented in the study are included in the article/Supplementary material, further inquiries can be directed to the corresponding author.
