# Supplementary material for: Effects of music-based interventions on rehabilitation outcomes after stroke: a systematic review and meta-analysis of randomized controlled trials
Source: Front Neurol. 2026 Aug 28;17:1907080. doi: 10.3389/fneur.2026.1907080 (PMC13561795; doi:10.3389/fneur.2026.1907080)
Supplement: Supplementary file 1 [file Data_Sheet_1.DOCX]

**Appendix:**

**Table S1** Specific search strategies for electronic databases

| **Database** | **Search Query** | **Results** |
| --- | --- | --- |
|  | | |
| **Web of science** | #1 TS=(Stroke OR Strokes OR "Cerebrovascular Accident" OR "Cerebrovascular Accidents" OR "Cerebral Stroke" OR "Cerebral Strokes" OR "Stroke, Cerebral" OR "Strokes, Cerebral" OR "Cerebrovascular Apoplexy" OR "Apoplexy, Cerebrovascular" OR "Vascular Accident, Brain" OR "Brain Vascular Accident" OR "Brain Vascular Accidents" OR "Vascular Accidents, Brain" OR "Cerebrovascular Stroke" OR "Cerebrovascular Strokes" OR "Stroke, Cerebrovascular" OR "Strokes, Cerebrovascular" OR Apoplexy OR CVAS OR "Acute Stroke" OR "Acute Strokes" OR "Strokes, Acute" OR "Cerebrovascular Accident, Acute" OR "Acute Cerebrovascular Accident" OR "Acute Cerebrovascular Accidents" OR "Cerebrovascular Accidents, Acute")  #2 TS=(Music Therapy OR "Therapy, Music" OR Music)  #3 #1AND#2 | **778** |
|  |  |  |
| **PubMed** | (("Stroke"[Mesh]) OR (Strokes[Title/Abstract] OR Cerebrovascular Accident[Title/Abstract] OR Cerebrovascular Accidents[Title/Abstract] OR Cerebral Stroke[Title/Abstract] OR Cerebral Strokes[Title/Abstract] OR Stroke, Cerebral[Title/Abstract] OR Strokes, Cerebral[Title/Abstract] OR Cerebrovascular Apoplexy[Title/Abstract] OR Apoplexy, Cerebrovascular[Title/Abstract] OR Vascular Accident, Brain[Title/Abstract] OR Brain Vascular Accident[Title/Abstract] OR Brain Vascular Accidents[Title/Abstract] OR Vascular Accidents, Brain[Title/Abstract] OR Cerebrovascular Stroke[Title/Abstract] OR Cerebrovascular Strokes[Title/Abstract] OR Stroke, Cerebrovascular[Title/Abstract] OR Strokes, Cerebrovascular[Title/Abstract] OR Apoplexy[Title/Abstract] OR CVA (Cerebrovascular Accident[Title/Abstract]) OR CVAs (Cerebrovascular Accident[Title/Abstract]) OR Stroke, Acute[Title/Abstract] OR Acute Stroke[Title/Abstract] OR Acute Strokes[Title/Abstract] OR Strokes, Acute[Title/Abstract] OR Cerebrovascular Accident, Acute[Title/Abstract] OR Acute Cerebrovascular Accident[Title/Abstract] OR Acute Cerebrovascular Accidents[Title/Abstract] OR Cerebrovascular Accidents, Acute[Title/Abstract])) AND (("Music Therapy"[Mesh]) OR (Therapy, Music[Title/Abstract] OR Music[Title/Abstract])) | **244** |
|  |  |  |
| **Scopus** | ( TITLE-ABS-KEY ( Stroke OR Strokes OR "Cerebrovascular Accident" OR "Cerebrovascular Accidents" OR "Cerebral Stroke" OR "Cerebral Strokes" OR "Stroke, Cerebral" OR "Strokes, Cerebral" OR "Cerebrovascular Apoplexy" OR "Apoplexy, Cerebrovascular" OR "Vascular Accident, Brain" OR "Brain Vascular Accident" OR "Brain Vascular Accidents" OR "Vascular Accidents, Brain" OR "Cerebrovascular Stroke" OR "Cerebrovascular Strokes" OR "Stroke, Cerebrovascular" OR "Strokes, Cerebrovascular" OR "Apoplexy OR Stroke, Acute" OR "Acute Stroke" OR "Acute Strokes" OR "Strokes, Acute" OR "Cerebrovascular Accident, Acute" OR "Acute Cerebrovascular Accident" OR "Acute Cerebrovascular Accidents" OR "Cerebrovascular Accidents, Acute" ) AND TITLE-ABS-KEY ( Music Therapy OR "Therapy, Music" OR Music ) ) | **1248** |
|  |  |  |
| **Embase** | #1 'cerebrovascular accident'/exp OR 'cerebrovascular accident' OR (cerebrovascular AND ('accident'/exp OR accident)) OR 'accident, cerebrovascular':ti,ab,kw OR 'acute cerebrovascular lesion':ti,ab,kw OR 'acute focal cerebral vasculopathy':ti,ab,kw OR 'acute stroke':ti,ab,kw OR 'apoplectic stroke':ti,ab,kw OR 'apoplexia':ti,ab,kw OR 'apoplexy':ti,ab,kw OR 'blood flow disturbance, brain':ti,ab,kw OR 'brain accident':ti,ab,kw OR 'brain attack':ti,ab,kw OR 'brain blood flow disturbance':ti,ab,kw OR 'brain insult':ti,ab,kw OR 'brain insultus':ti,ab,kw OR 'brain vascular accident':ti,ab,kw OR 'cerebral apoplexia':ti,ab,kw OR 'cerebral insult':ti,ab,kw OR 'cerebral stroke':ti,ab,kw OR 'cerebral vascular accident':ti,ab,kw OR 'cerebral vascular insufficiency':ti,ab,kw OR 'cerebro vascular accident':ti,ab,kw OR 'cerebrovascular arrest':ti,ab,kw OR 'cerebrovascular failure':ti,ab,kw OR 'cerebrovascular injury':ti,ab,kw OR 'cerebrovascular insufficiency':ti,ab,kw OR 'cerebrovascular insult':ti,ab,kw OR 'cerebrum vascular accident':ti,ab,kw OR 'cryptogenic stroke':ti,ab,kw OR 'cva':ti,ab,kw OR 'insultus cerebralis':ti,ab,kw OR 'ischaemic seizure':ti,ab,kw OR 'ischemic seizure':ti,ab,kw OR 'stroke':ti,ab,kw OR 'thrombotic stroke':ti,ab,kw OR 'cerebrovascular accident':ti,ab,kw  #2 'music therapy'/exp OR 'music therapy' OR (('music'/exp OR music) AND ('therapy'/exp OR therapy)) OR 'melotherapy':ti,ab,kw OR 'therapy, music':ti,ab,kw OR 'music therapy':ti,ab,kw  #3 #1 AND #2 | **1011** |
|  |  |  |
| **Cochrane**  **Library** | #1 MeSH descriptor: [Stroke] this term only )  #2 (Acute Strokes OR Strokes, Acute OR Acute Cerebrovascular Accidents OR Acute Cerebrovascular Accident OR Acute Stroke OR Cerebrovascular Accidents, Acute OR Cerebrovascular Accident, Acute OR Stroke, Acute OR Stroke, Cerebral OR Cerebrovascular Apoplexy OR Cerebrovascular Stroke OR CVA (Cerebrovascular Accident) OR Brain Vascular Accidents OR Vascular Accidents, Brain OR Cerebrovascular Strokes OR Strokes OR Apoplexy, Cerebrovascular OR Strokes, Cerebrovascular OR Vascular Accident, Brain OR Stroke, Cerebrovascular OR Brain Vascular Accident OR Apoplexy OR CVAs (Cerebrovascular Accident) OR Cerebral Strokes OR Strokes, Cerebral OR Cerebrovascular Accident OR Cerebral Stroke OR Cerebrovascular Accidents):ti,ab,kw  #3 #1 OR #2  #4 MeSH descriptor: [Music Therapy] explode all trees  #5 Synonyms OR Therapy, Music  #6 #4 OR #5  #7 #3 AND #6 | **201** |
|  |  |  |
| **ProQuest** | summary(("Stroke" OR Strokes OR "Cerebrovascular Accident" OR "Cerebrovascular Accidents" OR "Cerebral Stroke" OR "Cerebral Strokes" OR "Stroke, Cerebral" OR "Strokes, Cerebral" OR "Cerebrovascular Apoplexy" OR "Apoplexy, Cerebrovascular" OR "Vascular Accident, Brain" OR "Brain Vascular Accident" OR "Brain Vascular Accidents" OR "Vascular Accidents, Brain" OR "Cerebrovascular Stroke" OR "Cerebrovascular Strokes" OR "Stroke, Cerebrovascular" OR "Strokes, Cerebrovascular" OR Apoplexy OR "CVA (Cerebrovascular Accident)" OR "CVAs (Cerebrovascular Accident)" OR "Stroke, Acute" OR "Acute Stroke" OR "Acute Strokes" OR "Strokes, Acute" OR "Cerebrovascular Accident, Acute" OR "Acute Cerebrovascular Accident" OR "Acute Cerebrovascular Accidents" OR "Cerebrovascular Accidents, Acute")) AND summary(("Music Therapy" OR "Therapy, Music" OR Music)) | 557 |
|  |  |  |
| **CINAHL** | (SU Stroke OR T1 (Strokes OR "Cerebrovascular Accident" OR "Cerebrovascular Accidents" OR "Cerebral Stroke" OR "Cerebral Strokes" OR "Stroke, Cerebral" OR "Strokes, Cerebral" OR "Cerebrovascular Apoplexy" OR "Apoplexy, Cerebrovascular" OR "Vascular Accident, Brain" OR "Brain Vascular Accident" OR "Brain Vascular Accidents" OR "Vascular Accidents, Brain" OR "Cerebrovascular Stroke" OR "Cerebrovascular Strokes" OR "Stroke, Cerebrovascular" OR "Strokes, Cerebrovascular" OR "Apoplexy OR Stroke, Acute" OR "Acute Stroke" OR "Acute Strokes" OR "Strokes, Acute" OR "Cerebrovascular Accident, Acute" OR "Acute Cerebrovascular Accident" OR "Acute Cerebrovascular Accidents" OR "Cerebrovascular Accidents, Acute") OR AB (Strokes OR "Cerebrovascular Accident" OR "Cerebrovascular Accidents" OR "Cerebral Stroke" OR "Cerebral Strokes" OR "Stroke, Cerebral" OR "Strokes, Cerebral" OR "Cerebrovascular Apoplexy" OR "Apoplexy, Cerebrovascular" OR "Vascular Accident, Brain" OR "Brain Vascular Accident" OR "Brain Vascular Accidents" OR "Vascular Accidents, Brain" OR "Cerebrovascular Stroke" OR "Cerebrovascular Strokes" OR "Stroke, Cerebrovascular" OR "Strokes, Cerebrovascular" OR "Apoplexy OR Stroke, Acute" OR "Acute Stroke" OR "Acute Strokes" OR "Strokes, Acute" OR "Cerebrovascular Accident, Acute" OR "Acute Cerebrovascular Accident" OR "Acute Cerebrovascular Accidents" OR "Cerebrovascular Accidents, Acute")) AND (SU Music Therapy OR T1 ( "Therapy, Music" OR Music) OR AB ( "Therapy, Music" OR Music)) | **280** |

**Table S2** Characteristics of stroke stage and baseline cognitive function in the included studies

| Study | Time since stroke | Stroke stage | Baseline cognitive assessment | Cognitive eligibility criteria |
| --- | --- | --- | --- | --- |
| Thaut et al. 1997 | Within 3 months | Acute | Not reported | Able to complete gait training |
| Schauer et al. 2003 | Median 44 days | Subacute | Not reported | Independent walking |
| Thaut et al. 2007 | EG: 21.3 days CG: 22.3 days | Subacute | Not reported | Able to complete gait training |
| Cha et al. 2014 | ≥6 months | Chronic | MMSE-K ≥20 | MMSE-K ≥20 |
| Suh et al. 2014 | Not reported | Chronic | MMSE reported at baseline only | MMSE-K ≥24 |
| Tong et al. 2015 | EG: 5.4 ± 4.8 months  CG: 5.3 ± 4.1 months | Not reported | Not reported | No cognitive impairment or aphasia |
| Song et al. 2016 | EG: 12.30 ± 3.4 months  CG: 14.75 ± 6.0 months | Chronic | MMSE ≥24 | MMSE ≥24 |
| Poćwierz-Marciniak et al. 2017 | Approximately 1 month | Early post-stroke rehabilitation | MMSE and FAB | MMSE ≥27  FAB ≥16 |
| Jia et al. 2017 | 2 weeks to 3 months | Subacute | Not reported | Participants were required to be conscious, clinically stable, and free from evident cognitive impairment |
| Bunketorp-Käll et al. 2017 | 10 months to 5 years | chronic | Using the Barrow Neurological Institute Screen for Higher Cerebral Functions | Individuals with cognitive were excluded |
| Raglio et al. 2017 | After the acute phase. | Post-acute | MMSE ≥18 | MMSE ≥18 |
| Jia et al. 2018 | 2 weeks to 3 months | Subacute | Not reported | Participants were required to be conscious, clinically stable, and without obvious cognitive impairment |
| Zhou et al. 2018 | EG:45.18 ±11.38 d  CG:46.75 ± 9.32 d | Subacute | Not reported | Patients with aphasia or cognitive impairment were excluded |
| Fujioka et al. 2018 | ≥6 months | Chronic | Baseline MoCA (MST): 24.7 ± 2.8 | Participants had to have no dementia or cognitive |
| Study | Time since stroke | Stroke stage | Baseline cognitive assessment | Cognitive eligibility criteria |
| Grau-Sanchez et al. 2018 | Less than 6 months | Subacute | MMSE >24 | MMSE >24 |
| Hu et al. 2021 | 2 weeks to 6 months | Subacute | Not reported | Patients with cognitive impairment were excluded |
| Wang et al. 2021 | Stroke onset 2–12 days | Acute | Not reported | Participants were required to be conscious, clinically stable, without cognitive impairment or hearing impairment |
| Raglio et al. 2021 | Range 12–180 days | Subacute | Not reported | Not reported |
| Wang Y et al. 2021 | Disease duration ≤3 months; participants had already regained walking ability for ≥1 month | Subacute | Not reported | Participants were required to have normal cognition. |
| Ding et al. 2023 | Participants were 6 months to 1 year onset | Chronic | Not reported | Not reported |
| Fan et al. 2024 | Time since stroke not explicitly restricted. | Post-stroke | Cognitive function was assessed using the Montreal Cognitive Assessment (MoCA) | Participants were able to cooperate with the intervention |

**Table S3** Risk of bias analysis of each included study

| Study,year | selection bias（random sequence generation） | selection bias  （allocation concealment） | performance bias | detection bias | attrition bias | reporting bias | other bias |
| --- | --- | --- | --- | --- | --- | --- | --- |
| Thaut et al. 1997 | unclear | unclear | high | low | low | low | low |
| Schauer et al. 2003 | low | unclear | high | unclear | low | low | low |
| Thaut et al. 2007 | low | low | high | low | low | low | low |
| Cha et al. 2014 | low | low | high | unclear | low | low | low |
| Suh et al, 2014 | low | low | high | unclear | low | low | low |
| Tong et al. 2015 | low | unclear | high | unclear | low | low | low |
| Song et al. 2016 | unclear | unclear | high | unclear | low | low | low |
| Pocwierz-Marciniak 2017 | low | unclear | high | unclear | low | low | low |
| Jia et al.2017 | low | unclear | high | low | low | low | low |
| Bunketorp-Käll et al.2017 | low | low | high | low | low | low | low |
| Raglio et al.2017 | low | unclear | high | low | low | low | low |
| Jia et al.2018 | low | unclear | high | low | low | low | low |
| Zhou et al.2018 | low | unclear | high | unclear | low | low | low |
| Fujioka et al. 2018 | low | low | high | low | low | low | low |
| Grau‑Sánchez et al. 2018 | low | low | high | low | low | low | low |
| Study,year | selection bias（random sequence generation） | selection bias  （allocation concealment） | performance bias | detection bias | attrition bias | reporting bias | other bias |
| Hu et al.2021 | low | unclear | high | unclear | low | low | low |
| Wang et al. 2021 | low | unclear | high | unclear | low | low | low |
| Raglio A et al. 2021 | low | low | high | low | low | low | low |
| Wang Y et al. 2021 | low | unclear | high | unclear | low | low | low |
| Ding et al. 2023 | unclear | unclear | high | unclear | low | low | low |
| Fan et al. 2024 | low | low | low | low | low | low | low |

**Table S4** Methodological quality assessment of the included studies using the PEDro scale

| Study | Q1 | Q2 | Q3 | Q4 | Q5 | Q6 | Q7 | Q8 | Q9 | Q10 | Q11 | Total (0-10) | Study quality |
| --- | --- | --- | --- | --- | --- | --- | --- | --- | --- | --- | --- | --- | --- |
| Thaut et al. 1997 | Yes | Yes | No | Yes | No | No | Yes | Yes | Yes | Yes | Yes | 7 | Good |
| Schauer et al. 2003 | Yes | Yes | No | Yes | No | No | No | Yes | Yes | No | Yes | 5 | Acceptable |
| Thaut et al. 2007 | Yes | Yes | Yes | Yes | No | No | Yes | Yes | Yes | Yes | Yes | 8 | Good |
| Cha et al. 2014 | Yes | Yes | Yes | Yes | No | No | No | Yes | Yes | Yes | Yes | 7 | Good |
| Suh et al, 2014 | Yes | Yes | Yes | Yes | No | No | No | Yes | Yes | Yes | Yes | 7 | Good |
| Tong et al. 2015 | Yes | Yes | No | Yes | No | No | No | Yes | No | Yes | Yes | 5 | Acceptable |
| Song et al. 2016 | Yes | Yes | No | Yes | No | No | No | Yes | Yes | Yes | Yes | 6 | Good |
| Pocwierz-Marciniak 2017 | Yes | Yes | No | Yes | No | No | No | Yes | Yes | Yes | Yes | 6 | Good |
| Jia et al.2017 | Yes | Yes | No | Yes | No | No | Yes | Yes | Yes | Yes | Yes | 7 | Good |
| Bunketorp-Käll et al.2017 | Yes | Yes | Yes | Yes | No | No | Yes | Yes | Yes | Yes | Yes | 8 | Good |
| Raglio et al.2017 | Yes | Yes | No | Yes | No | No | Yes | Yes | Yes | Yes | Yes | 7 | Good |
| Jia et al.2018 | Yes | Yes | No | Yes | No | No | Yes | Yes | Yes | Yes | Yes | 7 | Good |
| Zhou et al.2018 | Yes | Yes | No | Yes | No | No | No | Yes | Yes | Yes | Yes | 6 | Good |
| Fujioka et al. 2018 | Yes | Yes | No | Yes | No | No | Yes | Yes | Yes | Yes | Yes | 7 | Good |
| Grau‑Sánchez et al. 2018 | Yes | Yes | Yes | Yes | No | No | Yes | Yes | No | Yes | Yes | 7 | Good |
| Hu et al.2021 | Yes | Yes | No | Yes | No | No | No | Yes | Yes | Yes | Yes | 6 | Good |
| Study | Q1 | Q2 | Q3 | Q4 | Q5 | Q6 | Q7 | Q8 | Q9 | Q10 | Q11 | Total (0-10) | Study quality |
| Wang et al. 2021 | Yes | Yes | No | Yes | No | No | No | Yes | Yes | Yes | Yes | 6 | Good |
| Raglio A et al. 2021 | Yes | Yes | No | Yes | No | No | Yes | Yes | Yes | Yes | Yes | 7 | Good |
| Wang Y et al. 2021 | Yes | Yes | No | Yes | No | No | No | Yes | Yes | Yes | Yes | 6 | Good |
| Ding et al. 2023 | Yes | Yes | No | Yes | No | No | No | Yes | No | Yes | Yes | 5 | Acceptable |
| Fan et al. 2024 | Yes | Yes | Yes | Yes | Yes | No | Yes | Yes | No | Yes | Yes | 8 | Good |

**Figure S1** Funnel plot of upper limb motor function (n=8)

**Figure S2** Funnel plot of gait speed (n=6)

**Figure S3** Funnel plot of balance function (n=4)

**Figure S4** Funnel plot of lower limb motor function (n=4)

**Figure S5** Funnel plot of ADL (n=7)

**Figure S6** Funnel plot of QoL (n=8)
